# Supplementary material for: Mechanisms governing the pioneering and redistribution capabilities of the non-classical pioneer PU.1
Source: Nat Commun. 2020 Jan 21;11:402. doi: 10.1038/s41467-019-13960-2 (PMC6972792; doi:10.1038/s41467-019-13960-2)
Supplement: Supplementary file 7 — Source data [file 41467_2019_13960_MOESM7_ESM.zip › Source_Data/Figure6/Figure6B_MotifScanOutput/ETS1_mutPU.1/homerResults/motif43.similar.html]

motif43

## Information for motif43

C
T
G
A
T
G
C
A
T
C
A
G
T
C
G
A
A
C
G
T
T
C
A
G
C
T
A
G
T
G
A
C
A
C
T
G
A
T
C
G
  
Reverse Opposite:  

T
A
G
C
T
G
A
C
A
C
T
G
G
A
T
C
A
G
T
C
T
G
C
A
A
G
C
T
A
G
T
C
A
C
G
T
G
A
C
T
  

|  |  |
| --- | --- |
| p-value: | 1e-33 |
| log p-value: | -7.685e+01 |
| Information Content per bp: | 1.571 |
| Number of Target Sequences with motif | 369.0 |
| Percentage of Target Sequences with motif | 2.74% |
| Number of Background Sequences with motif | 394.3 |
| Percentage of Background Sequences with motif | 1.12% |
| Average Position of motif in Targets | 97.9 +/- 59.1bp |
| Average Position of motif in Background | 103.7 +/- 63.9bp |
| Strand Bias (log2 ratio + to - strand density) | -0.2 |
| Multiplicity (# of sites on avg that occur together) | 1.03 |
| Motif File: | file (matrix) reverse opposite |

### Similar de novo motifs found

|  |  |  |  |  |  |  |  |
| --- | --- | --- | --- | --- | --- | --- | --- |
| Rank | Match Score | Redundant Motif | P-value | log P-value | % of Targets | % of Background | Motif file |
| 1 | 0.925 | C T G A C T G A T A C G T C G A C A G T A T C G C T A G A T G C A C T G T A C G A G T C | 1e-24 | -57.288005 | 2.33% | 1.03% | motif file (matrix) |
| 2 | 0.833 | C T G A C T G A T C A G T C G A C A G T A T C G A C T G A G T C C A T G T A C G T A G C A C T G T A G C G A T C | 1e-24 | -56.385716 | 1.76% | 0.67% | motif file (matrix) |
| 3 | 0.863 | T G A C G T C A C G T A T C A G C T G A A C G T A C T G A C T G G T A C A C T G T A C G A G T C | 1e-24 | -56.024928 | 1.68% | 0.63% | motif file (matrix) |
| 4 | 0.753 | A C G T A T G C G T C A T C G A T C G A T C G A C G A T T C A G T A C G G A T C C A T G A C T G G A T C | 1e-21 | -50.056153 | 2.98% | 1.56% | motif file (matrix) |
